# ‘Now you can see me, now you don’t’: seeking the invisible lung nodule

**DOI:** 10.1093/icvts/ivad048

**Published:** 2023-03-17

**Authors:** Thrasyvoulos P Michos, Sotirios I Sterpis, Periklis I Tomos, Emmanouil I Kapetanakis

**Affiliations:** Department of Thoracic Surgery, National and Kapodistrian University of Athens, Athens, Greece; School of Medicine, National and Kapodistrian University of Athens, Athens, Greece; Department of Thoracic Surgery, National and Kapodistrian University of Athens, Athens, Greece; Department of Thoracic Surgery, National and Kapodistrian University of Athens, Athens, Greece

**Keywords:** Ground glass opacity, Lung nodule, Video thoracoscopy, Ultrasound

*Dura est manus cirurgi, sed sanans.*

The hand of the surgeon is hard, but healing.

Walter Map, (1135–1210), British writer

Since minimally invasive surgery (MIS) such as video-assisted thoracoscopic surgery (VATS) was introduced over 2 decades ago, it has revolutionized the thoracic surgery field. MIS can offer many advantages such as reduced anaesthesia and hospitalization time, less tissue damage and pain, decreased intraoperative blood loss, lower risk of postoperative infection and complications, better cosmetic outcome and faster recovery compared to traditional open technique [1–4]. When conducted together with an enhanced recovery after surgery protocol, VATS may produce better results with a significantly reduced length of hospital stay and postoperative complication incidence [5].

However, VATS is definitely not a panacea and it actually has a number of technical drawbacks [1, 2, 4]. One of the main ones is the loss of 3D vision [2, 4]. The image the surgeon sees to operate by is captured, transmitted and projected onto a 2D monitor resulting in the loss of depth judgement and a reduced visual field [4]. There is also a limited range of motion due to the longer, rigid, less ergonomic instrumentation used and the fixed port positions [1, 4]. This can result in a longer operative time and also increased associated cost due to the use of more expensive surgical equipment and staplers [4].

Another significant limitation of MIS is the lack of tactile sense during tool—tissue interaction that severely impairs the surgeon’s ability to control the applied forces—and thus can cause unintentional damage or additional trauma to healthy tissues [1]. Tactile sensation is crucial not only for safely manipulating organs, structures, tissues and sutures but also for getting reliable information about the thickness, stiffness and texture of tissues through palpation, which is crucial for tumour detection [2].

Recent advances and more widespread application of computational tomography (CT) including the use of low-dose high-resolution CT protocols have allowed for the early detection of small pulmonary nodules (PNs) and improved early-stage lung cancer screening [5, 6]. VATS is routinely utilized for the early diagnosis and treatment of these PNs [5, 6]. However, small, deeply positioned PNs can be difficult to visualize and locate during VATS. This is due to the previously mentioned restrictions in the optical field and mainly the absence of tactile feedback since the fixed and limited access available means thoracic surgeons cannot easily utilize finger palpation as they would during open surgery. The lack of palpation makes VATS resection of deep-seated PNs and/or ground glass opacities (GGOs) particularly difficult. Factors such as size, density and distance to the pleural surface can influence the nodule detection rate. Therefore, a number of studies have reported that up to 63% of patients undergoing VATS to resect PNs <10 mm in diameter or >5 mm from the pleura ultimately have to undergo conversion to thoracotomy [5]. Trying to reduce these conversion rates, many studies have explored the use of preoperative and intraoperative localization techniques to guide surgeons during VATS resection of PNs [5]. Some of the most frequently utilized techniques for localization include the use of preoperative CT-guided tags such as mechanical coils or hook-wires, methylene blue or indigo-carmine dyes, contrast media and radiotracers such as technetium-99m [5, 6]. However, the preoperative labelling procedure is time consuming and may cause patient discomfort, complications such as pneumothorax or/and haemoptysis and radiation exposure, while dislodgement of the tag can often occur [7, 8].

In this article, Messina *et al.* [9] explore an alternative, less-invasive, more direct localizing technique for identifying GGO lesions during VATS. They utilized intraoperative ultrasound (IUS) to identify, locate and accurately describe 15 GGO lesions, which were subsequently thoracoscopically resected [9]. In this pilot study, IUS was able to effectively detect GGO lesions and delineate their features in terms of size, depth, solid component and surgical margins [9]. The authors therefore concluded that IUS can provide real-time feedback, reliably, repeatably and non-invasively to surgeons performing minimally invasive lung resections for the treatment of GGO lesions [9].

The use of IUS for detecting lung nodules is not novel! Almost 2 decades ago, Santambrogio *et al.* [10] utilized IUS to safely and effectively locate and resect non-palpable PNs in 18 patients. Recently, Taurchini *et al.* [11] utilized IUS to identify and locate suspicious lung nodules in 131 patients undergoing either thoracoscopic or open thoracotomy resection. The detection rate of lung nodules for ultrasound was 100% compared to digital palpation which was 94.66% [11].

However, in contrast to lung nodules, GGOs are small areas of hazy increased attenuation on CT that do not obscure underlying bronchial structures or vascular markings and often do not present with a solid, firm component. And herewith is their main difference compared to PNs, for their detection even with open palpation is extremely challenging! Nevertheless, high-frequency ultrasound probes such as the one used in Messina *et al.*’s study can overcome this disadvantage and have been shown to be very accurate locating small PNs of 20 mm or less at depths of up to 15 mm [8, 9].

Consequently, this report by Messina *et al.* can be considered a glimpse into the future because the authors, by utilizing high frequency IUS, were now able to not only quite effectively locate the GGOs but also to accurately define and characterize/describe them. Therefore, one may safely expect that as ultrasound resolution and technique improve further its applicability in thoracic surgery will probably become more widespread and thus the ‘*manus cirurgi*’ will end up replaced by ultrasound to effectuate ‘*sānantem*’!
